# Effectiveness of Telephone-Based Health Coaching for Patients with Chronic Conditions: A Randomised Controlled Trial

**DOI:** 10.1371/journal.pone.0161269

**Published:** 2016-09-15

**Authors:** Martin Härter, Jörg Dirmaier, Sarah Dwinger, Levente Kriston, Lutz Herbarth, Elisabeth Siegmund-Schultze, Isaac Bermejo, Herbert Matschinger, Dirk Heider, Hans-Helmut König

**Affiliations:** 1 Department of Medical Psychology, University Medical Centre Hamburg-Eppendorf, Hamburg, Germany; 2 Kaufmännische Krankenkasse—KKH, Hannover, Germany; 3 University Medical Centre Freiburg, Freiburg, Germany; 4 Department of Health Economics and Health Services Research, University Medical Centre Hamburg-Eppendorf, Hamburg, Germany; Kurume University School of Medicine, JAPAN

## Abstract

**Background:**

Chronic diseases, like diabetes mellitus, heart disease and cancer are leading causes of death and disability. These conditions are at least partially preventable or modifiable, e.g. by enhancing patients’ self-management. We aimed to examine the effectiveness of telephone-based health coaching (TBHC) in chronically ill patients.

**Methods and Findings:**

This prospective, pragmatic randomized controlled trial compares an intervention group (IG) of participants in TBHC to a control group (CG) without TBHC. Endpoints were assessed two years after enrolment. Three different groups of insurees with 1) multiple conditions (chronic campaign), 2) heart failure (heart failure campaign), or 3) chronic mental illness conditions (mental health campaign) were targeted. The telephone coaching included evidence-based information and was based on the concepts of motivational interviewing, shared decision-making, and collaborative goal setting. Patients received an average of 12.9 calls. Primary outcome was time from enrolment until hospital readmission within a two-year follow-up period. Secondary outcomes comprised the probability of hospital readmission, number of daily defined medication doses (DDD), frequency and duration of inability to work, and mortality within two years. All outcomes were collected from routine data provided by the statutory health insurance. As informed consent was obtained after randomization, propensity score matching (PSM) was used to minimize selection bias introduced by decliners. For the analysis of hospital readmission and mortality, we calculated Kaplan-Meier curves and estimated hazard ratios (HR). Probability of hospital readmission and probability of death were analysed by calculating odds ratios (OR). Quantity of health service use and inability to work were analysed by linear random effects regression models. PSM resulted in patient samples of 5,309 (IG: 2,713; CG: 2,596) in the chronic campaign, of 660 (IG: 338; CG: 322) in the heart failure campaign, and of 239 (IG: 101; KG: 138) in the mental health campaign. In none of the three campaigns, there were significant differences between IG and CG in time until hospital readmission. In the chronic campaign, the probability of hospital readmission was higher in the IG than in the CG (OR = 1.13; p = 0.045); no significant differences could be found for the other two campaigns. In the heart failure campaign, the IG showed a significantly reduced number of hospital admissions (-0.41; p = 0.012), although the corresponding reduction in the number of hospital days was not significant. In the chronic campaign, the IG showed significantly increased number of DDDs. Most striking, there were significant differences in mortality between IG and CG in the chronic campaign (OR = 0.64; p = 0.005) as well as in the heart failure campaign (OR = 0.44; p = 0.001).

**Conclusions:**

While TBHC seems to reduce hospitalization only in specific patient groups, it may reduce mortality in patients with chronic somatic conditions. Further research should examine intervention effects in various subgroups of patients, for example for different diagnostic groups within the chronic campaign, or duration of coaching.

**Trial Registration:**

German Clinical Trials Register DRKS00000584

## Introduction

Health care systems are faced with an increasing number of patients with chronic conditions such as cardiovascular, respiratory, or metabolic diseases due to the increasing prevalence of individual (e.g. unhealthy lifestyles) and environmental risk factors (e.g. air pollution), demographic changes (e.g. longer life expectancy), and medical progress. If not treated or managed adequately, chronic conditions result in a reduction of patients’ life quality [1, 2], and a high mortality accounting for nearly two thirds of deaths worldwide [3]. Consequently, chronic conditions account for most of health care expenditures [4, 5] and lost economic productivity [4, 6].

Telephone support for self-management or disease management is a promising approach to improve care for patients with chronic conditions [7–9]. Telephone-based health coaching (TBHC) aims to enhance patients’ self-management abilities by providing information for a better understanding of their conditions, to improve the ability to collaborate with health care providers, and to use goal setting related to disease management. Through early identification of patients with disease progressions, expensive health service use (e.g. hospital admissions) may be avoided [10]. A narrative review by Hutchison & Breckon [11] showed that patients receiving telephone coaching have various benefits, especially on clinical (e.g. physiological markers), behavioural (e.g. self-care regimen, adherence), and psychosocial (self-efficacy, mental health) outcomes. A subsequent review by Dennis et al. [12] came to similar conclusions, especially when coaching calls were regularly scheduled and their content was tailored to the patient´s individual needs, goals, and clinical situation. Both reviews also showed that the interventions were predominantly focusing on diabetes and cardiovascular diseases. A large heterogeneity of these interventions (e.g. several studies included tele-monitoring in addition to the telephone health coaching service) was observed, which makes it difficult to draw clear conclusions from the literature [11, 12].

Meanwhile, several additional studies have investigated the effects of generic telephone health coaching for patients with chronic conditions. The largest RCT in the US with 174,120 participants with various chronic conditions showed that generic telephone health coaching with elements of shared decision-making, patient information, self-management, and communication skills reduced hospitalization rates, surgical procedures, and health care costs [13]. However, the replication of the results was not successful until now, questioning the transferability of these results into routine clinical practice in other settings [10]. For instance, no effects on health services use were shown for participants compared with a matched comparison group [10, 14], or compared to a randomized control group [15].

In summary, the most coherent effects were found for telephone health coaching on psychosocial and self-reported outcomes like perceived health and self-efficacy. Study results in a real world setting are still ambiguous for economic effects. More research is needed to determine the cost-effectiveness of generic telephone health coaching services for patients with various chronic conditions [12]. Finally, evidence is needed with regard to the transferability of the trial effects conducted in the US, UK, or Australia to other health care systems.

### Objectives

The aim of this study was to evaluate the effectiveness of a telephone-based health coaching intervention (TBHC) for patients with chronic conditions in Germany. The primary hypothesis was that TBHC will extend the time period until the patient’s hospital readmission compared to usual care without TBHC.

## Methods

### Trial design

This prospective, pragmatic randomised controlled trial compared participants of TBHC to usual care. The rationale, study design and statistical analysis procedures have been published [16]. The Hamburg Medical Chamber Ethics Committee approved the study protocol. The protocol of this trial and supporting CONSORT checklist are available as supporting information: see S1 and S2 Files. The study complies with the Helsinki Declaration (last update 2013); written informed consent was obtained from all participants prior to enrolment. The trial was registered nine months late due to unexpected delays in the course of contract negotiations with the funding institution (KKH) and resulting problems with timely recruitment of scientific staff.

### Participants

Study participants were recruited between June 2010 and October 2011, had to be at least 18 years old, insured with the “Kaufmännische Krankenkasse (KKH)” (a nationwide statutory health insurance company with 1.8 million insured persons in Germany), and diagnosed with one or more of the following chronic conditions (based on the principal ICD-10 codes documented in the routine data sets recorded by the health insurance): diabetes type 2, coronary artery disease, hypertension, heart failure, asthma, chronic obstructive pulmonary disease (COPD), chronic depression or schizophrenia. The allocation of patients to one of the campaigns was based on the principal diagnosis within the routine data set. For patients with type 2 diabetes, hypertension or coronary artery diseases a risk score for hospital readmission was calculated based on a logistic regression model. The model was developed using stepwise logistic regression. The final regression model included following variables: 43 ICD-10 codes, sex, age, annual income, living area (rural /urbanized areas), hospital stays, hospital days and hospital costs within the last 12 months, and 13 ATC codes (Anatomical Therapeutic Chemicals). This model has been validated by analyzing the receiver operating characteristic curve (ROC) [17] and the positive predictive value [18] and showed valid predictions. If the risk score predicted a probability of hospital readmission of more than 50%, patients were included in the study (see S1 Table).

Based on various variables from routine data a logistic regression model was designed to predict hospital readmission using SPSS 19. These variables comprise e.g. health care costs, ICD-diagnoses, age and gender. This model has been validated by analyzing the receiver operating characteristic curve (ROC) [17] and the positive predictive value [18] and showed valid predictions.

Patients were excluded if they had insufficient German language skills or were not able to read or use a phone.

### The telephone-based health coaching intervention

The TBHC was developed originally by Health Dialog Inc. for chronically ill patients to enhance health behaviour change [13, 19]. Communication techniques like motivational interviewing, individual and collaborative goal setting, and shared decision-making were important components [13, 19]. This program has been adapted to the German health care system in 2007 and, subsequently, it was widely implemented by the KKH. A first analysis regarding acceptance indicated a generally very positive appraisal by the participants [20]. The intervention was tailored to important chronic conditions that are in need of similar self-management strategies:

1. “chronic campaign” for patients with type 2 diabetes, hypertension, asthma, COPD, and/or coronary artery disease;
2. “heart failure campaign” for patients with heart failure;
3. “mental health campaign” for patients with depression or schizophrenia.

More detailed information on the topics addressed by the intervention can be found as supporting information: see S3 File.

Coaching was conducted by 20 experienced nurses in two call centres located in Munich and Halle/Saale. They were supervised 2–4 times per year by two experienced supervisors from the project group (MH, IB).

The coaching approach was divided in 3 phases:

- Phase 1 (“welcome call”): The first call was for getting to know each other and for gathering information about the chronic condition, its severity, clinical parameters, health behaviour, and medication. The most important health issues and the motivation for change were assessed.
- Phase 2 (“orientation phase”): First goals were agreed upon, and the coach increased the insuree’s motivation for change. More information about the patient’s knowledge about his condition and coping with it was gathered.
- Phase 3 (“maintenance phase”): The coach recalled the agreements made in the last call, fostered the motivation for change, acknowledged success and set new goals together with the insurant. Symptoms and adherence to medication were continuously monitored, information material was sent out. Topics were necessary and useful medical check-ups, information on prevention, nutrition and diet, exercise, and stress management (see appendix 2).

The minimum frequency was defined as one telephone contact every six weeks with a maximum intervention duration of one year, which was extended to a maximum of two years in specific patients who needed further support. Specific intervention manuals for the coaches regarding different situations (e.g. for vaccination programs), available topics and accessible information materials provided support for the coaches. The coaches were further assisted by a health platform (netdoktor.de) providing evidence-based and up-to-date health information. NetDoktor is a health portal written and edited by health professionals, certified by HONcode (www.hon.ch) and related to specific criteria (www.afgis.de), two quality certifications for reliable online health information. Today, NetDoktor is one of the most visited health platforms in the German speaking world. Data on the coaching process, individual goal setting, medication, and clinical parameters (e.g. Hb_A1c_ and blood pressure) was recorded by an electronic documentation system. Written patient information for specific conditions, medication plans and weight-control tables could be sent to the participants. Additionally, patients in the heart failure campaign got a booster call. This call was only used to get data on the maintenance of actions (e.g. weighing and medication adherence), without any coaching intervention.

The control group received no coaching, but usual health care.

### Study outcomes

The primary outcome was the time from enrolment until the first hospital readmission during a two-year follow-up period. The secondary outcomes included the probability of hospital readmission, number of hospital admissions, number of hospital days, number of daily defined doses (DDD) of medication, frequency and duration of inability to work, as well as mortality. Calculation of duration of inability to work is based on the individual summarized duration of inability to work per study period (two years). Information on the frequency of inability to work is calculated from summing up the number of single episodes of inability to work per participant in the study period (two years).

All outcomes were analysed based on pseudonymised routine (claims) data collected and provided by the KKH, which were available for the complete randomized sample. The data contained information on all contacts with the health care system (including ICD-10 codes; operations and procedure key code–OPS, the German equivalent to the American procedure coding system—PCS), medication, and inability to work. The KKH assembled and pseudonymized the routine data. Disease-specific outcomes like disease-related hospital admission were disregarded due to comparability between the campaigns and due to the high trial complexity. Patient-reported outcomes like quality of life, depression and anxiety will be published separately as the sample differs due to different response rates.

### Randomisation

To provide the necessary comparability between intervention and control group we used a stratified random allocation design that was based on sociodemographic values with a 4:1 allocation ratio. The stratification variables differed in the campaigns: For the “heart failure campaign” the allocation parameters were amount of hospital days (0 to 30, 31 to 100, more than 100) and sex, for the “chronic campaign” allocation parameters were “hospital cases” (1, 2 or more than 2) and age (18 to 40, 41 to 60, older than 60) as previous data analyses showed that those were the most influential parameters on hospital costs.

Yet, due to ethical reasons, randomization had to be conducted before obtaining informed consent; thus the randomised intervention group consisted of coaching participants and decliners. The health insurance had to avoid the fact that they would not have been able to offer health coaching to all informed insures if informed consent would have been asked before. Subsequent to randomisation, members of the intervention group received a postal invitation to participate in the TBHC and an additional acquisition call. Insurees were included as participants, when they had sent back the informed consent and the confirmation of participation. If they did not send back the required confirmation, they were grouped as decliners. Members of the control group did not receive an invitation, but only the informed consent form for participating in the study.

### Statistical analysis

#### Propensity score matching

For each of the three campaigns participants were compared to insurees in the control group (CG). Potential selection bias was minimized by propensity score matching (PSM), which balanced baseline characteristics of patients in the IG and CG. Based on routine data from a 12-month pre-period, a linear propensity score was calculated using the following matching variables: age, participation in disease management program, employment status, marital status, federal state, early retirement, eligibility for statutory sick pay, costs of rehabilitation, hospital costs, outpatient physician and non-physician costs, medication costs, defined daily dose (DDD), costs of non-physician outpatient services and medical supplies, as well as the Elixhauser Index based on ICD codes [21] for days of disability, inpatient and outpatient visits. The Elixhauser Index [22] was developed for application in large administrative data sets and defines 31 conditions based on ICD-9 or ICD-10 codes [21]. For the definition of the 31 conditions, only secured ICD diagnosis from pooled outpatient, inpatient and rehabilitation data were used. The Elixhauser score is calculated by assigning specific weights to the 31 comorbidities if present. It has a maximum range from -19 to +89 [23]. A systematic review comparing the suitability of different comorbidity measures for administrative data found the Elixhauser to be the best available measure so far [24]. This is particularly true for the measurement of long-term mortality (>30 days). Participants were matched exactly according to information on sex, study centre, and study campaign (i.e. matching was not conducted separately by campaign but exact matching for the study campaign variable was performed instead). Nearest neighbour matching with a ratio of 1:4 was applied. The chosen caliper was 0.1. Controls were drawn with replacement. The PSM-procedure was conducted in R (Version 3.0.2) using the statistical package MatchIt (2.4–21) [25, 26]. Vectors indicating the membership to one of the study groups and weights were exported to STATA 13.0, where the analysis for inferential statistics was conducted. The balance before and after matching was checked by means of Quantile-Quantile-Plots and the so-called Percent Balance Improvement (PBI) [25]

#### Analysis

The sample was analysed using three approaches. Intention-to-Treat-I (ITT-I) followed the initial randomisation and resulted in coaching participants and decliners in the intervention group. In Intention to-treat-II analysis (ITT-II) the decliners were dropped from the intervention group. Finally, a per-protocol approach was conducted. Because of likely biased results in ITT-I due to included IG-decliners and in the per-protocol analysis because of including only those patients who completed the treatment, we focus on the presentation of results from the ITT-II approach. This approach can be regarded as most “realistic”. The other approaches served as sensitivity analysis and are not presented in detail.

For the analysis of the primary outcome (time until hospital readmission) as well as mortality (time to death), we calculated Kaplan-Meier curves and estimated hazard ratios from proportional hazard models. Probability of hospital readmission and probability of death were analysed by calculating odds ratio (OR). Quantity of health service use and inability to work was analysed by linear fixed effects regression models. As opposed to conventional PSM, the existence of a pre-period and a follow-up allowed us to control for latent heterogeneity in covariates by differencing out time-invariant additive selection bias through the application of the difference-in-difference method. The difference-in-difference (DD) is defined as follows:

$$\tau_{DD}=E[Y_{1}^{T}-Y_{0}^{T}|T_{1}=1]-E[Y_{1}^{C}-Y_{0}^{C}|T_{1}=1]$$

Given two-time-points with a pre-period (*t* = 0) and a follow-up (*t* = 1) the respective outcomes for the treatment group and the controls in time *t* hereby are denoted by $Y_{t}^{T}$ and $Y_{t}^{C}$. This way the DD will compare the treatment group and controls in terms of outcome changes over time in relation to the outcomes observed for the pre-period. The DD allows to compare both study groups with respect to temporal changes under consideration of the existing baseline differences of the respective outcomes. In regression analysis, the DD was applied by calculating the interaction term between membership to the IG and the study time (pre-period vs. follow-up). Thereby analytical weight variables stemming from the matching ratio of 1:4 were applied in fixed effects regression models using STATA 13.0. The level of significance was set at α = 0.05. As the campaigns differ regarding to the target population as well as the provided intervention, we evaluated all campaigns in one regression but separated the DDs for the specific campaigns by including additional three-way interactional effects between time, study group and study campaign.

## Results

### Patient flow

**Fig 1:**
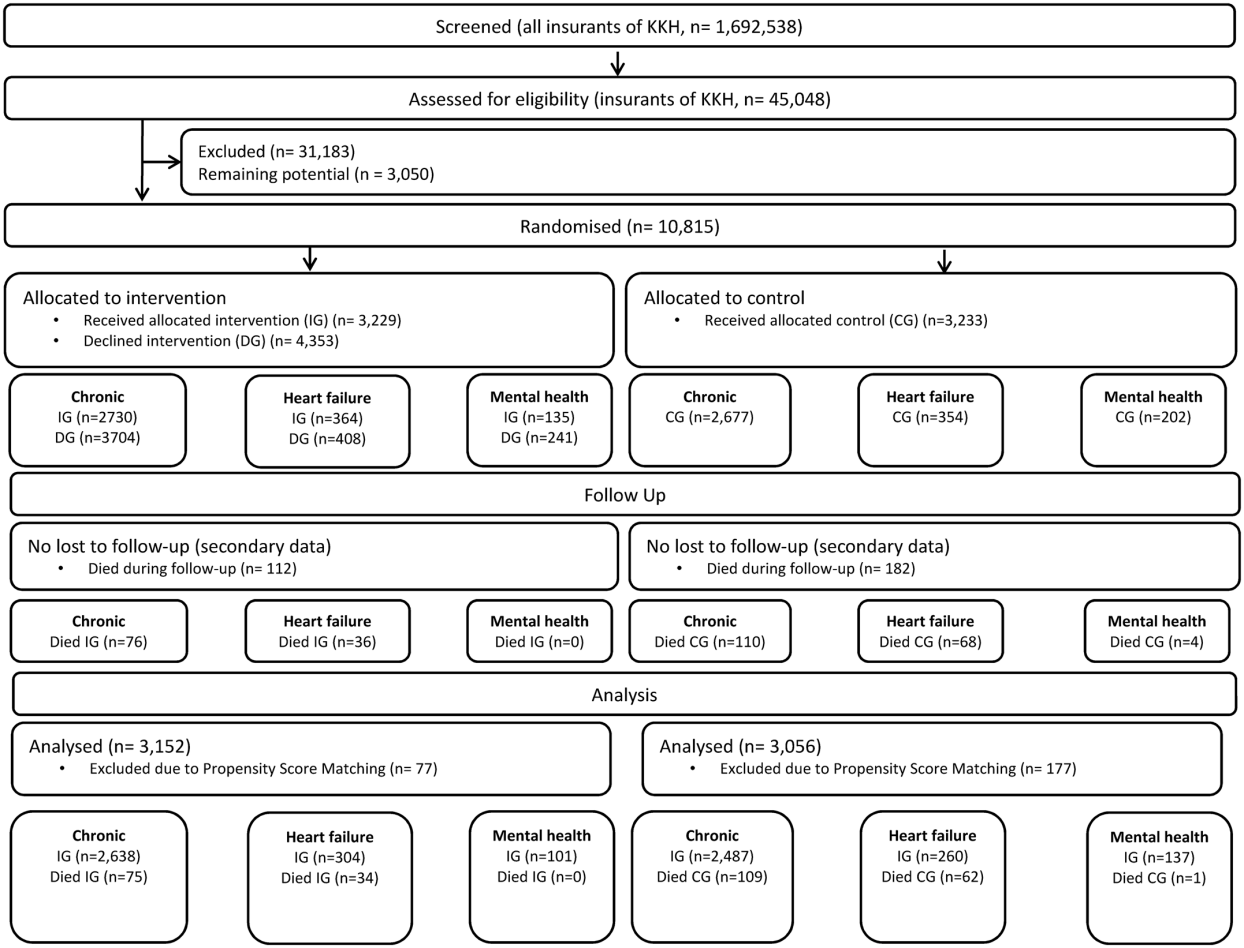
Patient flow.

Patient selection resulted in N = 10,815 patients eligible for the coaching intervention. Randomisation allocated to the IG: 6,434 patients in the chronic campaign, 772 in the heart failure campaign, and 376 in the mental health campaign, of whom 2,730 (42.4%), 364 (47.2%) and 135 (35.9%), respectively, consented to participate in the intervention. PSM resulted in patient samples of 5,309 (IG: 2,713; CG: 2,596) in the chronic campaign, 660 (IG: 338; CG: 322) in the heart failure campaign, and 239 (IG: 101; CG: 138) in the mental health campaign to be used for the primary analysis (Fig 1).

### Patient characteristics

Within the three campaigns, participants (IG) and controls (CG) were very similar with respect to age, sex, and comorbidity measured by the Elixhauser Index at baseline (Table 1). However, there were marked differences between campaigns, with patients in the mental health campaign being younger, more likely female, and more often single and less comorbid than in the other campaigns. The frequencies of diseases as defined by the Elixhauser Index are provided as supporting information (see S3 Table). Furthermore, differences between IG and CG in the number of hospital admissions, number of hospital days, number of DDDs, as well as frequency and duration of inability to work were small within campaigns during the 12-month pre-period. Yet, hospital days and inability to work were more frequent in the mental health campaign, while the mean number DDD was higher in the chronic campaign and the heart failure campaign.

**Table 1:** Sample Characteristics at Baseline, Pre- and Post-Matching.

PSM^a^		Chronic campaign				Heart failure campaign				Mental health campaign			
		CG^b^		IG^c^		CG^b^		IG^c^		CG^b^		IG^c^	
		Mean	(SD)	Mean	(SD)	Mean	(SD)	Mean	(SD)	Mean	(SD)	Mean	(SD)
Age	Pre-PSM	69.05	(8.52)	69.31	(7.89)	71.04	(10.26)	70.59	(9.91)	44.79	(11.97)	45.71	(11.18)
	Post-PSM	69.25	(8.27)	69.02	(7.91)	71.27	(10.17)	70.49	(9.67)	46.60	(11.02)	46.35	(10.62)
Elixhauser Comorbidity Index (inpatient)	Pre-PSM	4.21	(5.79)	4.19	(5.87)	14.26	(6.68)	14.01	(6.41)	-1.48	(2.87)	-1.99	(2.52)
	Post-PSM	4.21	(5.79)	4.15	(5.91)	13.79	(6.40)	13.68	(6.28)	-1.59	(2.54)	-2.34	(2.38)
Elixhauser Comorbidity Index (outpatient)	Pre-PSM	6.43	(7.56)	6.55	(7.69)	12.81	(8.51)	12.40	(8.41)	-1.04	(4.34)	-1.11	(4.54)
	Post-PSM	6.75	(7.67)	6.74	(7.67)	13.00	(8.82)	12.49	(8.26)	-1.15	(4.84)	-1.84	(3.91)
Elixhauser Comorbidity Index (inability to work)	Pre-PSM	2.11	(3.54)	2.19	(3.81)	7.16	(3.16)	6.68	(4.41)	-2.28	(1.76)	-2.41	(1.62)
	Post-PSM	2.16	(4.15)	2.09	(3.38)	6.37	(3.51)	7.80	(3.88)	-2.39	(1.92)	-3.00	(1.47)
Female (%)	Pre-PSM	1558	(58.20)	3719	(57.80)	174	(49.15)	383	(49.61)	140	(69.31)	264	(70.21)
	Post-PSM	1516	(58.40)	1554	(57.28)	161	(50.00)	160	(47.34)	107	(77.54)	81	(80.20)
Hospital admissions	Pre-PSM	1.18	(1.19)	1.22	(1.19)	2.06	(1.51)	1.94	(1.18)	2.08	(0.89)	2.10	(0.90)
	Post-PSM	1.19	(1.21)	1.22	(1.20)	2.00	(1.45)	1.96	(1.24)	2.04	(0.89)	2.13	(0.91)
Hospital days	Pre-PSM	11.58	(53.52)	11.69	(50.76)	22.60	(22.62)	21.83	(48.07)	80.05	(40.23)	87.02	(44.65)
	Post-PSM	12.50	(66.59)	11.38	(44.82)	20.94	(18.54)	19.25	(15.78)	78.13	(37.47)	85.83	(42.71)
DDD^a^	Pre-PSM	2265.97	(1282.54)	2266.05	(1247.18)	2401.31	(1426.85)	2373.34	(1431.26)	872.38	(896.58)	853.23	(791.00)
	Post-PSM	2274.35	(1274.03)	2292.56	(1225.46)	2417.49	(1446.64)	2385.14	(1397.24)	941.57	(912.29)	869.80	(667.68)
Cases of inability to work	Pre-PSM	0.41	(1.24)	0.35	(1.09)	0.24	(0.77)	0.37	(1.11)	1.19	(1.42)	1.31	(1.96)
	Post-PSM	0.37	(1.18)	0.35	(1.11)	0.20	(0.72)	0.31	(1.02)	0.96	(1.28)	1.36	(1.61)
Days of inability to work	Pre-PSM	15.19	(69.53)	14.95	(69.09)	20.44	(83.28)	30.09	(108.08)	91.73	(143.10)	121.21	(172.54)
	Post-PSM	15.95	(73.51)	16.38	(70.60)	17.36	(73.45)	23.48	(91.39)	83.20	(129.85)	106.83	(142.55)

^a^ = Propensity score matching

^b^ = Control group

^c^ = Intervention group

### Intervention

The intervention was carried out as planned. The mean amount of calls was 12.9 (SD = 7.3) for all participants, with the lowest quartile (Q1) receiving up to 7 calls, a median of 13 calls and an upper quartile receiving up to 18 calls. This implies an interquartile range of 11. Participants in the chronic campaign received an average of 12.8 calls (SD = 6.5), in the heart failure campaign 14.0 calls (SD = 8.4) and in the mental health campaign 13.7 calls (SD = 14.4). The calls lasted for an average of 20.6 minutes (SD = 8.4) for all participants, 21.2 minutes (SD = 8.4) for the chronic campaign, 17.5 minutes (SD = 7.3) for the heart failure campaign and 17.5 minutes (SD = 7.1) for the mental health campaign. About 7% of the participants received nutrition counselling, (7.1% of the chronic campaign, 4.4 of the heart failure campaign and 11.3% of the mental health campaign).

The main goals the coaches aimed at were the understanding of illness (in 58.7% of the participants), colon cancer screening (46.8%), vaccination against pneumococcae (44.7%), self-monitoring of blood pressure (32.6%), preparation and discussion of physician visit (21.2%), and weight reduction (17.6%). All goal categories are available as supporting information: see S2 Table.

### Primary outcome

#### Time to hospital readmission

**Fig 2:**
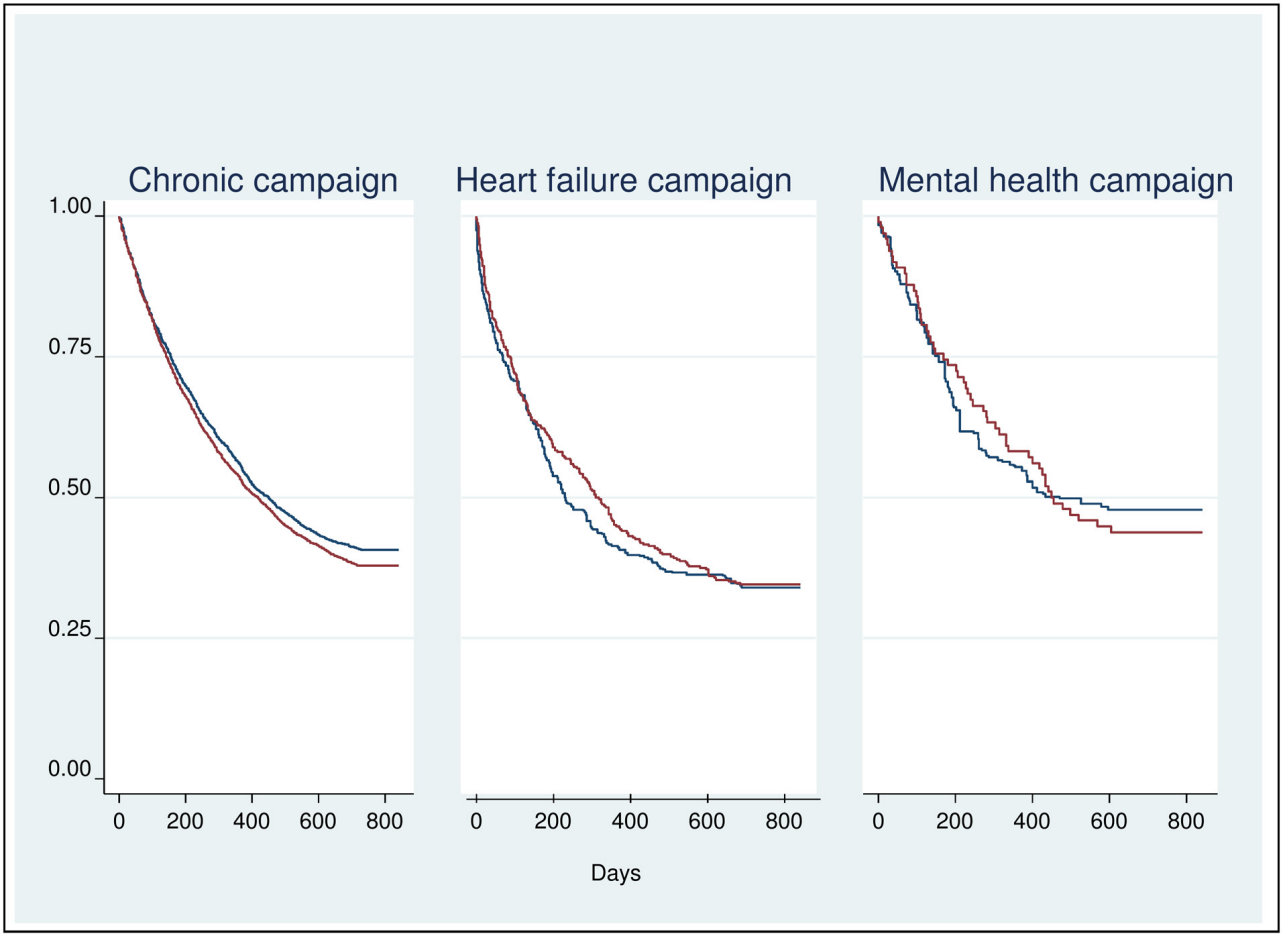
Effects of telephone-based health coaching on time until hospital readmission. Kaplan-Meier curves showing the proportion of individuals without hospital readmission over time (red curve = intervention group; blue curve = control group).

In none of the campaigns, IG and CG differed significantly regarding the time to readmission to hospital during the two-year follow-up period (Fig 2). The hazard ratio (HR) for the chronic campaign was HR = 1.07 (p = 0.083), for the heart failure campaign HR = 0.94 (p = 0.558) and for the mental health campaign HR = 1.01 (p = 0.968).

### Secondary outcomes

#### Hospital use, medication and inability to work

In all groups, more than 50% of the patients were readmitted to hospital during follow-up. In the chronic campaign, the odds of hospital readmission during follow-up was higher in the IG (1.65) than in the CG (1.46), resulting in an odds ratio of 1.13 (p = 0.045). No significant differences were found for the other two campaigns. Linear random effects difference-in-difference regression analysis of hospital use, medication and inability to work showed a significantly reduced number of hospital admission in the IG of the heart failure campaign (-0.41; p = 0.012); yet, the corresponding reduction in the number of hospital days (-6.17; p = 0.249) did not reach the level of significance (Table 2).

**Table 2:** Effects of telephone-based health coaching on healthcare utilization: Results of linear fixed effects difference-in-difference regression models (ITT-II).

	Chronic campaign	Heart failure campaign	Mental health campaign
	Diff-in-Diff^b^ (SE)	Diff-in-Diff^b^ (SE)	Diff-in-Diff^b^ (SE)
Hospital admissions	0,10 (0,06)	-0,41 (0,22)*	0,26 (0,26)
Hospital days	3,09 (2,27)	-6,17 (4,18)	-0,07 (10,68)
DDD^a^	155,03 (49,66)***	298,68 (194,31)	-162,42 (198,47)
Cases of inability to work	0,01 (0,04)	-0,04 (0,07)	-0,08 (0,28)
Days of inability to work	-0,25 (2,57)	-3,67 (7,12)	-16,39 (21,30)

^a^ = Daily defined doses of medication

^b^ = Difference in difference

* p<0.05;

** p<0.01;

***p<0.001

Furthermore, the number of DDDs increased significantly by 155.03 (p = 0.001) in the IG of the chronic campaign. All other regression results were not statistically significant which implies that there were no differences with respect to the frequency and duration of inability to work. The descriptives of health care utilization can be found in Table 3.

**Table 3:** Descriptive statistics of health care utilization (Post-Matching).

Time		Chronic campaign		Heart failure campaign		Mental health campaign	
		CG^b^	IG^c^	CG^b^	IG^c^	CG^b^	IG^c^
		Mean (SD)	Mean (SD)	Mean (SD)	Mean (SD)	Mean (SD)	Mean (SD)
Hospital cases	T_0_	1,19 (1,21)	1,22 (1,20)	2,00 (1,45)	1,96 (1,24)	2,04 (0,89)	2,13 (0,91)
	T_2_	1,54 (1,81)	1,67 (1,98)	2,35 (2,63)	1,90 (2,19)	1,34 (1,60)	1,69 (1,96)
Hospital days	T_0_	12,50 (66,59)	11,38 (44,82)	20,94 (18,54)	19,25 (15,78)	78,13 (37,47)	85,83 (42,71)
	T_2_	14,39 (40,25)	16,37 (44,90)	28,24 (51,13)	20,39 (44,07)	36,85 (59,27)	44,49 (74,70)
DDD^a^	T_0_	2274,35 (1274,03)	2292,56 (1225,46)	2417,49 (1446,64)	2385,14 (1397,24)	941,57 (912,29)	869,80 (667,68)
	T_2_	4613,37 (2604,22)	4786,60 (2534,59)	4896,82 (3197,76)	5163,14 (2841,91)	2287,92 (2350,35)	2053,73 (1601,67)
Cases of inability to work	T_0_	0,37 (1,18)	0,35 (1,11)	0,20 (0,72)	0,31 (1,02)	0,96 (1,28)	1,36 (1,61)
	T_2_	0,48 (1,75)	0,47 (1,77)	0,22 (0,90)	0,30 (1,16)	1,41 (2,30)	1,73 (2,61)
Days of inability to work	T_0_	15,95 (73,51)	16,38 (70,60)	17,36 (73,45)	23,48 (91,39)	83,20 (129,85)	106,83 (142,55)
	T_2_	12,22 (60,06)	12,41 (59,63)	3,94 (31,09)	6,39 (38,70)	34,85 (93,33)	42,10 (96,22)

^a^ = Daily defined doses of medication

^b^ = Control group

^c^ = Intervention group

T_0_ = Baseline; T_2_ = 2 years after baseline

#### Mortality

**Fig 3:**
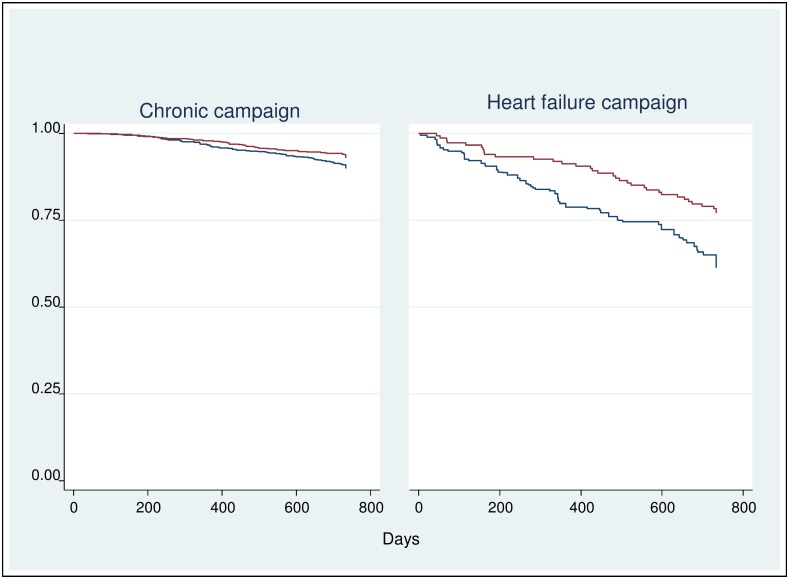
Effects of telephone-based health coaching on mortality. Kaplan-Meier survival curves (red curve = intervention group; blue curve = control group).

In the chronic campaign, 75 (2.8%) patients of the IG died during follow-up compared to 109 (4.2%) patients of the CG, resulting in an OR = 0.64 (p = 0.005) and a hazard ratio of HR = 0.64 (p = 0.005). In the heart failure campaign, 34 (10.1%) patients of the IG died compared to 62 (19.3%) patients of the CG, resulting in an OR = 0.44 (p = 0.001) and a hazard ratio HR = 0.47 (p = 0.001). In the mental health campaign only 1 patient of the CG died (Fig 3). Thus, the calculation of HR, OR and Kaplan-Meier survival curve for the mental health campaign is impossible.

### Sensitivity analysis

Results of the ITT-I-approach differ considerably from the ITT-II-analysis (Table 4). This can be seen as an indicator for the successful balancing of the data and the validity of the results from ITT-II.

**Table 4:** Effects of telephone-based health coaching on healthcare utilization: Results of linear fixed effects difference-in-difference regression models (ITT-I).

	Chronic campaign	Heart failure campaign	Mental health campaign
	Diff-in-Diff^b^ (SE)	Diff-in-Diff^b^ (SE)	Diff-in-Diff^b^ (SE)
Hospital admissions	-0.01 (0.05)	-0.14 (0.13)	-0.12 (0.18)
Hospital days	0.50 (1.54)	-3.53 (4.30)	-9.15 (5.85)
DDD^a^	43.78 (39.91)	304.42 (111.38) ***	72.15 (151.37)
Cases of inability to work	-0.06 (0.03)**	-0.00 (0.09)	-0.15 (0.12)
Days of inability to work	-2.30 (2.11)	-5.19 (5.90)	-29.22 (8.02)***

^a^ = Daily defined doses of medication

^b^ = Difference in difference

* p<0.05;

** p<0.01;

*** p<0.001

In contrast, the per-protocol analysis differed only slightly (Table 5).

**Table 5:** Effects of telephone-based health coaching on healthcare utilization: Results of linear fixed effects difference-in-difference regression models (per protocol).

	Chronic campaign	Heart failure campaign	Mental health campaign
	Diff-in-Diff^b^ (SE)	Diff-in-Diff^b^ (SE)	Diff-in-Diff^b^ (SE)
Hospital admissions	0.13* (0.06)	-0.35 (0.19)	0.14 (0.30)
Hospital days	1.83 (1.74)	-4.56 (5.34)	1.16 (8.60)
DDD^a^	124.98** (48.33)	329.13* (148.01)	21.43 (238.36)
Cases of inability to work	-0.01 (0.04)	-0.04 (0.12)	0.00 (0.19)
Days of inability to work	0.29 (2.46)	-6.89 (7.54)	-19.70 (12.14)

^a^ = Daily defined doses of medication

^b^ = Difference in difference

* p<0.05;

** p<0.01;

*** p<0.001

However, due to the small sample size, even very small changes in effects can result in changed significances. For instance, within the heart failure campaign, hospital admissions are significant in ITT-II, but not in the per-protocol analysis (vice versa in the chronic campaign).

## Discussion

### Statement of principal findings

Telephone health coaching is expected to improve chronic disease management, which in turn is expected to avoid corresponding hospital admission. We compared insurees of a German statutory health insurance selected by predictive risk modelling with various chronic conditions who obtained one year of TBHC within three different campaigns (chronic conditions, heart failure, mental health) to a randomized and matched control group using routine data. The coaching did not result in extending the time period until the patient`s next hospital readmission compared with usual care during 24 months of follow-up in general as expected in our primary study hypothesis. Probability of hospital readmission was instead found to be significantly higher for participants in chronic campaign. However, for participants in the heart failure campaign, but not for participants of the chronic or mental health campaign, the number of hospital admissions was significantly reduced at 24 months follow-up, but no differences could be observed with regard to number of hospital days. We could not confirm the hypothesis that telephone health coaching reduces hospital admissions. However, participants of the TBHC intervention were significantly less likely to die within the 2-year follow-up period than participants of the CG, both in the chronic and particularly the heart failure campaign, which can be regarded as a very important patient-relevant effect of the TBHC.

### Strengths and weaknesses

A strength of the study is its large sample size, as we were able to include over 2,700 insured patients in the IG. This trial is surely one of the largest randomized controlled trials of TBHC to date. Furthermore, we could use routine data from the statutory health insurance company for all study participants, thus avoiding problems of non-response and potential sample or systematic biases [27]. The data were not influenced by non-response or recall-bias, as they were collected directly at the healthcare providers. The validity of routine data in Germany is not proven, yet studies show that the ICD diagnoses are valid in about 97% for heart failure patients [19, 28]. A further strength of this study lies in the high treatment fidelity of the TBHC due to the structured, manual-based approach with regular supervision and quality assurance measures. The study was conducted in routine care with a long 24-month follow-up period, which enhances the external validity or generalizability of the results. Comparable studies [10, 13] used only 12 months follow-up periods. Finally, important chronic disease groups were included, i.e. patients with frequent chronic conditions, heart failure or a chronic mental illness.

There are some limitations to the work presented: diagnoses for chronic conditions like depression, as data are documented primarily for accounting purposes, could be biased by financial incentives. Additionally, general practitioners are not well trained in making psychiatric diagnoses. Consequently, there might be a bias concerning the validity of these diagnoses. Another limitation is the lack of insurees’ clinical data, which makes it difficult to assess disease severity and other characteristics and risk factors that might have influenced the disease course, thus potentially influencing results and interpretations.

A major cause for potential bias lies in the fact, that we could enrol patients only after randomisation due to ethical reasons of the health insurance. A selection bias could have resulted due to the high percentage of 57% of decliners to participation. The participants of the TBHC group could have been less impaired or may have a higher health consciousness than the control group (“healthy user bias”), which in turn could have resulted in lower mortality rates. We tried to reduce this risk of bias by establishing a control group via propensity score matching, balancing baseline characteristics of patients in the IG and CG, and by differencing out time-invariant additive selection bias through the application of the difference-in-difference method. As a result of the matching process, observable differences between intervention and control group were eliminated. However, not all potentially relevant variables were available within our routine data set, so the matching procedure may have missed out other relevant variables. For example, information on socioeconomic status was not available from routine data, but has a potential influence on health status and service use. Therefore, we cannot completely exclude a healthy user bias, but we are confident that we ruled out the most significant impact. Moreover, the chronic campaign was composed of a very heterogeneous group of patients with different diagnoses with various health-related problems. Therefore, the coaching intervention might have different effects for different subgroups of patients within this campaign, which should be a focus of subsequent analysis. Another possible source of bias could result from the fact that the population consists of insured individuals with a higher socioeconomic status than the general population. However, for all citizens, the health care benefits of the compulsory insurance are fundamentally equal and defined by law, and almost every citizen in Germany is insured for health care. The German health care system is based on self-government and solidarity and is contribution-financed. The majority of the population (approx. 89%) are mandatory members of the public health insurance system. The remaining 11% have private health insurance [15].

### Strengths and weaknesses in relation to other studies

With the exception to the number of hospital admission in the heart failure campaign, our results did not confirm the hypothesis of TBHC reducing hospital use. This result is consistent with two systematic reviews. In the review by Hutchinson and colleagues [11], the authors originally referenced three studies with favourable effects on hospital use. However, each of the three studies combined TBHC with telemonitoring, so it remains unclear to which of the components of the intervention the effect can be ascribed. Dennis and colleagues [12] included 30 studies evaluating telephone health coaching for various chronic conditions. For telephone health coaching similar to our intervention (following a structured script with scheduled calls), none of six identified studies showed reductions in hospital or health service use. Finally, in two recent cohort studies with matched controls, hospital admissions did not change [14] or even increased in the intervention group [10] which is comparable to our findings in the chronic campaign and may be attributed to a better symptom monitoring by the coaches. Yet, in these trials using non-randomly chosen matched comparison groups [10, 14], unobserved confounding may have biased the results. Furthermore, these studies mainly focused on disease management (guiding patients on medical care or social services), less on coaching patients to manage their conditions. Additionally, often the intended dose could not be delivered, resulting in infrequent contacts between health coach and patients and a low treatment intensity.

To our knowledge, up to date only two studies came to different conclusions. A large randomized controlled trial showed decreases in hospital admissions [13], and a cohort study with matched controls showed lower inpatient, lower outpatient, and total expenditures [29]. The differences between these results might be explained by differences in the coaching intervention and the study design. For example, a more intense use of patient decision aids for previously identified preference-sensitive treatment options, as realized in the study by Wennberg et al. [13], could positively influence intervention effects [30]. Moreover, the study of Jonk and colleagues did not use a randomization procedure to generate the control group, but used only a comparison group generated by PSM, and the sample size was significantly smaller than in our trial [29].

Focusing only on patients with chronic heart failure, Inglis and colleagues provided a systematic review and meta-analysis including 41 studies on structured telephone support or non-invasive home tele-monitoring [31]. Their results showed that structured telephone support reduced all-cause mortality and heart failure-related hospitalisations. Moreover, they reported positive effects of the interventions on health-related quality of life, heart failure knowledge and self-care behaviours. No effect of the interventions was found on the risk of all-cause hospitalisations.

The observed reduction of mortality in the chronic campaign was similar to that achieved in one previous trial [32], but contradicts analysis with regard to (in-hospital) mortality within a more recent study [10]. However, the study of Alkema and colleagues had a much smaller sample size, thus limiting validity of the results. In the study of Steventon et al [10], mortality was not used as primary or secondary outcome, but analysed only in the context of a sensitivity analysis.

### Unanswered questions and future research

Although we tried to avoid the possible bias due to enrolment after randomization by propensity score matching, differences between intervention and control group are still possible. Therefore, future trials should incorporate a randomization strategy after obtaining informed consent—thereby minimizing potential sources of bias—with subsequent intention-to-treat analysis. If such a design should be difficult to realizes, a further possibility might be to use an appropriate modification of a post-randomized consent (Zelen´s) design [33]. Furthermore, detailed economic evaluation could estimate possible cost savings in relation to the costs of the intervention [16]. Further analysis will also provide insight into patient-reported outcomes like quality of life, changes in health behaviour and risk factors, as well as health literacy and patient activation [16]., It will also be important to examine intervention effects in various subgroups of patients, for example for different diagnostic groups within the chronic campaign, or duration of coaching. This could provide additional insight into the question, which specific patient and condition characteristics are associated with greater benefits from the telephone coaching intervention and thus inform decisions about how to prioritize implementation efforts. Moreover, the more pronounced effects in the heart failure campaign provide evidence that effectiveness of telephone health coaching might be increased when tailored to specific target groups. Finally, it would be interesting to find out whether the quantity of calls is associated with better outcomes.

## Conclusion

Based on the results of our study and previous research, it seems that TBHC has only limited effects on hospital use, medication, and ability to work. Most important however, our results show that TBHC may reduce mortality as a strong patient-relevant outcome. Possible explanations include a better disease management and a higher awareness when to seek additional medical support by the participants, thus avoiding acute exacerbations of chronic conditions. The significant and patient-relevant reduction in mortality may be a strong argument to further invest in the development, implementation, and evaluation of promising TBHC interventions.

## Supporting Information

S1 File Study Protocol. Telephone-based health coaching for chronically ill patients: study protocol for a randomized controlled trial.
(PDF)

S2 File Consort checklist. CONSORT 2010 checklist of information to include when reporting a randomised trial.
(DOC)

S3 File TIDieR checklist. Template forIntervention Description and Replication checklist.
(DOCX)

S1 Table Regression model. Regression model to identify patients with a risk of rehospitalisation higher than 50%.
(DOCX)

S2 Table Coaching goals. Descriptives of coaching goals.
(DOCX)

S3 Table Frequencies of diseases. Frequencies of diseases* as defined by the Elixhauser Index, at Baseline (Post-PSM), by campaign.
(DOCX)

## Abbreviations

TBHC: Telephone based health coaching
CG: Control group
IG: Intervention group
DDD: Daily defined medication doses
PSM: Propensity score matching
OR: Odds ratio
HR: Hazard ratio

## References

[1] Murray CJL, Vos T, Lozano R, Naghavi M, Flaxman AD, Michaud C, et al Disability-adjusted life years (DALYs) for 291 diseases and injuries in 21 regions, 1990–2010: a systematic analysis for the Global Burden of Disease Study 2010. Lancet. 2012;380(9859):2197–223. 10.1016/S0140-6736(12)61689-4 23245608

[2] Jia H, Zack MM, Thompson WW. The effects of diabetes, hypertension, asthma, heart disease, and stroke on quality-adjusted life expectancy. Value Health. 2013;16(1):140–7. 10.1016/j.jval.2012.08.2208 23337225 PMC4590983

[3] Lozano R, Naghavi M, Foreman K, Lim S, Shibuya K, Aboyans V, et al Global and regional mortality from 235 causes of death for 20 age groups in 1990 and 2010: a systematic analysis for the Global Burden of Disease Study 2010. Lancet. 2012;380(9859):2095–128. 10.1016/S0140-6736(12)61728-0 23245604 PMC10790329

[4] Bloom DE, Cafiero ET, Jané-Llopis E, Abrahams-Gessel S, Bloom LR, Fathima S., Feigl AB, et al The global economic burden of non-communicable diseases. Geneva: World Economic Forum, 2011.

[5] Centers for Medicare and Medicaid Services. Chronic conditions among Medicare beneficiaries, chartbook, 2012 edition 2012.

[6] DeVol R, Bedroussian A. An unhealthy America: the economic burden of chronic disease charting a new course to save lives and increase productivity and economic growth. Milken Institute, 2007.

[7] Sinnott C, Mc Hugh S, Browne J, Bradley C. GPs' perspectives on the management of patients with multimorbidity: systematic review and synthesis of qualitative research. BMJ Open. 2013;3(9):e003610 10.1136/bmjopen-2013-003610 24038011 PMC3773648

[8] Richards T. People with chronic disease should be encouraged to manage their care. BMJ. 2012;344. 10.1136/bmj.e2771 22511390

[9] Bauer UE, Briss PA, Goodman RA, Bowman BA. Prevention of chronic disease in the 21st century: elimination of the leading preventable causes of premature death and disability in the USA. Lancet. 2014;384(9937):45–52. 10.1016/S0140-6736(14)60648-6 24996589

[10] Steventon A, Tunkel S, Blunt I, Bardsley M. Effect of telephone health coaching (Birmingham OwnHealth) on hospital use and associated costs: cohort study with matched controls. BMJ. 2013;347:f4585 10.1136/bmj.f4585 23920348 PMC3805495

[11] Hutchison AJ, Breckon JD. A review of telephone coaching services for people with long-term conditions. J Telemed Telecare. 2011;17(8):451–8. 10.1258/jtt.2011.110513 22025743

[12] Dennis SM, Harris M, Lloyd J, Powell Davies G, Faruqi N, Zwar N. Do people with existing chronic conditions benefit from telephone coaching? A rapid review. Aust Health Rev. 2013;37(3):381–8. 10.1071/AH13005 23701944

[13] Wennberg DE, Marr A, Lang L, O'Malley S, Bennett G. A randomized trial of a telephone care-management strategy. N Engl J Med. 2010;363(13):1245–55. 10.1056/NEJMsa0902321 20860506

[14] Lin WC, Chien HL, Willis G, O'Connell E, Rennie KS, Bottella HM, et al The effect of a telephone-based health coaching disease management program on Medicaid members with chronic conditions. Med Care. 2012;50(1):91–8. 10.1097/MLR.0b013e31822dcedf 21993059

[15] Kim SE, Michalopoulos C, Kwong RM, Warren A, Manno MS. Telephone care management's effectiveness in coordinating care for Medicaid beneficiaries in managed care: a randomized controlled study. Health Serv Res. 2013;48(5):1730–49. 10.1111/1475-6773.12060 23557249 PMC3796111

[16] Dwinger S, Dirmaier J, Herbarth L, Konig HH, Eckardt M, Kriston L, et al Telephone-based health coaching for chronically ill patients: study protocol for a randomized controlled trial. Trials. 2013;14:337 10.1186/1745-6215-14-337 24135027 PMC4016132

[17] McClish DK. Analyzing a portion of the ROC curve. Med Decis Making. 1989;9(3):190–5. 2668680 10.1177/0272989X8900900307

[18] Altman DG, Bland JM. Statistics Notes: Diagnostic tests 2: predictive values. Bmj. 1994;309(6947):102. 8038641 10.1136/bmj.309.6947.102 PMC2540558

[19] Schubert I, Ihle P, Koster I. [Internal confirmation of diagnoses in routine statutory health insurance data: concept with examples and case definitions]. Gesundheitswesen. 2010;72(6):316–22. 10.1055/s-0030-1249688 20480460

[20] Härter M, Dwinger S, Seebauer L, Simon D, Herbarth L, Siegmund-Schultze E, et al Evaluation of telephone health coaching of German health insurants with chronic conditions. Health Educ J. 2013;72:622–34.

[21] Quan H, Sundararajan V, Halfon P, Fong A, Burnand B, Luthi JC, et al Coding algorithms for defining comorbidities in ICD-9-CM and ICD-10 administrative data. Med Care. 2005;43(11):1130–9. 16224307 10.1097/01.mlr.0000182534.19832.83

[22] Elixhauser A, Steiner C, Harris DR, Coffey RM. Comorbidity measures for use with administrative data. Med Care. 1998;36(1):8–27. 9431328 10.1097/00005650-199801000-00004

[23] van Walraven C, Austin PC, Jennings A, Quan H, Forster AJ. A modification of the Elixhauser comorbidity measures into a point system for hospital death using administrative data. Med Care. 2009;47(6):626–33. 10.1097/MLR.0b013e31819432e5 19433995

[24] Sharabiani MT, Aylin P, Bottle A. Systematic review of comorbidity indices for administrative data. Med Care. 2012;50(12):1109–18. 10.1097/MLR.0b013e31825f64d0 22929993

[25] http://imai.princeton.edu/research/files/matchit.pdf.

[26] http://gking.harvard.edu/matchit.

[27] Ritter PL, Stewart AL, Kaymaz H, Sobel DS, Block DA, Lorig KR. Self-reports of health care utilization compared to provider records. J Clin Epidemiol. 2001;54(2):136–41. 11166528 10.1016/s0895-4356(00)00261-4

[28] Hoffmann F, Pfannkuche M, Glaeske G. [How often are dates of prescription and dispensing of drugs correct in claims data?]. Bundesgesundheitsblatt. 2007;50(11):1418–23. 10.1007/s00103-007-0369-5 17999135

[29] Jonk Y, Lawson K, O'Connor H, Riise KS, Eisenberg D, Dowd B, et al How effective is health coaching in reducing health services expenditures? Med Care. 2015;53(2):133–40. 10.1097/MLR.0000000000000287 25588134

[30] Veroff D, Marr A, Wennberg DE. Enhanced support for shared decision making reduced costs of care for patients with preference-sensitive conditions. Health Aff. 2013;32(2):285–93. 10.1377/hlthaff.2011.0941 23381521

[31] Inglis SC, Clark RA, Dierckx R, Prieto‐Merino D, Cleland JG. Structured telephone support or non‐invasive telemonitoring for patients with heart failure. Cochrane Database Syst Rev. 2015;10:CD007228 10.1002/14651858.CD007228.pub3 26517969 PMC8482064

[32] Alkema GE, Wilber KH, Shannon GR, Allen D. Reduced mortality: the unexpected impact of a telephone-based care management intervention for older adults in managed care. Health Serv Res. 2007;42(4):1632–50. 17610441 10.1111/j.1475-6773.2006.00668.x PMC1955273

[33] Zelen M. A new design for randomized clinical trials. N Engl J Med. 1979;300(22):1242–5. 431682 10.1056/NEJM197905313002203

