# Supplementary material for: Effectiveness of Telephone-Based Health Coaching for Patients with Chronic Conditions: A Randomised Controlled Trial
Source: PLoS One. 2016 Sep 15;11(9):e0161269. doi: 10.1371/journal.pone.0161269 (PMC5025178; doi:10.1371/journal.pone.0161269)
Supplement: S3 File — Template forIntervention Description and Replication checklist. (DOCX) [file pone.0161269.s003.docx]

| **Item No** | | **Item** | **Response (all information is provided in this appendix 2)** |
| --- | --- | --- | --- |
| 1 | **Brief name** | Provide the name or a phrase that describes the intervention | A telephone-based health coaching for patients with long-term diseases |
| 2 | **Why** | Describe any rationale, theory, or goal of the elements essential to the intervention | Motivational interviewing; health behaviour change; individual, collaborative goal setting; shared decision-making  The TBHC intervention is tailored to certain chronic conditions that are in need of similar self-management strategies called campaigns:   1. “chronic campaign” for type 2 diabetes, hypertension and coronary artery disease, 2. “heart failure campaign” for heart failure 3. “mental health campaign” for depression and schizophrenia   Aim of the coaching is to foster knowledge about the chronic condition and its treatment, like clinical values, check-ups and medication. Also the coaching tried to enhance self-management strategies and health behaviours, like a healthy diet, exercise and vaccination. Techniques used to achieve those goals are motivational interviewing, behaviour counselling and shared decision-making. |
| 3 | **What** | Materials: Describe any physical or informational materials used in the intervention, including those provided to participants or used in intervention delivery or in training of intervention providers. Provide information on where the materials can be accessed (such as online appendix, URL) | **For the coaches:**  The telephone health coaching is manualised. For each campaign there is a similar, but tailored manual supporting the coaches to gather all necessary information and to remember all possible coaching topics. The manual can be inquired at Kaufmännische Krankenkasse, Hannover.  The coaches provide evidence-based, up-to-date information and are supported by the website www.netdoktor.de. Also they are supported by a computer software called “ProvidinCare®” that enables the coaches to document each call thoroughly.  **For the patients:**  The coaching was supported by information material and education leaflets for specific conditions, medication plans and weight-control tables. Most information material can be accessed at the website of Kaufmännische Krankenkasse, Hannover(27).  Examples for sent out material (depending on chronic condition)   - Generic information:   - Medication list (to fill out)   - Medication information (e.g. Insulin, Marcumar)   - “Health passport” (by German Heart Foundation (28))   - Information about clinical parameters (e.g. HbA1c, blood pressure, metabolic syndrome, diabetic feet)   - Information about critical situations (e.g. hyperglycaemia, hypoglycaemia, symptoms of stroke and heart attack, emergency calls)   - Health behaviours     - “Physically fit with diabetes”, “My diabetes exercise program”     - “My ideal weight”, “Wholesome foods”, “Low-salt diet”, “Weight diary”     - “Yes, I will quit smoking”     - “Vaccination means protection”     - “Taking action with… (Diabetes, Hypertension etc.)”     - Stress management   - Recommended check-ups   - Information about depression and anxiety - Additional material for “heart failure campaign”   - Starters package:     - “Knowing heart failure and being active”     - “My partner has heart failure”     - “My guidebook for living with heart failure”     - “My blood pressure diary”     - Information for physician - Additional material for “mental health campaign”   - - “Depression – more than a mood swing”     - There was no additional material for schizophrenia |
| 4 | **How** | Procedures: Describe each of the procedures, activities, and/or processes used in the intervention, including any enabling or support activities | First the patient was assigned to one campaign based on the eligibility criteria.  The telephone coaching is divided in 4 to 5 phases:   1. Acquisition    - Explanation of telephone coaching    - Asking whether patient would like to participate 2. Welcome    - Getting-to-know each other    - Gathering information about chronic conditions and its severity (e.g. NYHA-status, pulmonary function), clinical parameters (e.g. HbA_1c_, cholesterol, potassium, sodium, shortness of breath, sputum, peak flow results), health behaviours, medication, interests    - Identification of most important health issue    - Identification of motivation to change    - “heart failure campaign”: Scoring (see (9) “*Tailoring”*)    - “heart failure campaign”: Starters package (see (3) “*What*”: *health information*)    - “mental health campaign”: Therapeutic, social and trusted contacts    - “mental health campaign”: stabilising resources 3. Orientation phase    - Increasing motivation to change    - Gathering information about severity of chronic condition and adherence, knowledge about chronic condition and coping mechanisms    - Possibly first goal setting    - Verifying whether patients medication goes along with guidelines    - Additional in “heart failure campaign”: The correlation between heart failure and weight, measuring heart failure 4. Theme-centred coaching calls    - Recalling agreements of last call    - Talking about information leaflets / material    - Monitoring of symptoms    - Adherence and changes of medication    - Goal setting (SMART) and recalling    - Fostering relationship    - Motivating to change    - If applicable: Celebrating success    - Sending out materials (see (3) *“What”*: Sent out materials)    - Generic coaching topics (depending on condition, identified risks, tailored)      - Understanding of condition      - Heart attack and stroke      - Emergency plan      - Measuring blood pressure      - Check-up (Eyes, feet, kidney)      - nutrition / diet, metabolism, weight      - exercise      - anxiety and depression      - smoking cessation      - stress management      - vaccination (Pneumococcus, influenza)      - travel    - Additional topics for “heart failure campaign”      - Anticoagulation      - Control of fluid intake    - Topics for “mental health campaign”      - crisis management      - self-care      - alcohol abstinence      - drug abstinence      - stabilising social contacts      - day structure      - psychoeducation (relapse prophylaxis, negative emotions, stress reduction, early warning signs, activity plan, mood diary) 5. “heart failure campaign”: Booster call    - Checking on actions    - Checking whether patient still weighs himself    - Checking on medication adherence    - Gathering information about clinical parameters (e.g. NYHA-status, weight, blood pressure, pulse, potassium, sodium and creatinine) |
| 5 | **Who provided** | For each category of intervention provider (such as psychologist, nursing assistant), describe their expertise, background, and any specific training given | The coaches were nurses with work experience as well as one nutrition scientist and two coaches who have special expertise with psychiatric patients as psychiatry nurses. In 2007 two coaches have been trained to carry out the coaching by staff directly qualified in the United States by Health Dialog, the company that developed this TBHC in the United States. The coaches were supervised two to three times a year. The supervisors are MH, the leading manager of the project, medical doctor and licensed psychotherapist, and IBB, a licensed psychotherapist and expert in quality management. |
| 6 | **How** | Describe the modes of delivery (such as face to face or by some other mechanism, such as internet or telephone) of the intervention and whether it was provided individually or in a group | The TBHC was be delivered by phone. There was be no possible face-to-face session. The TBHC is provided individually. |
| 7 | **Where** | Describe the type(s) of location(s) where the intervention occurred, including any necessary infrastructure or relevant features | The TBHC has been provided by phone directly to the patients’ home. The coaches were located in two areas in Germany: one team was operating from Munich, coaching patients living in the south of Germany, and one from Halle/Saale, responsible for patients from northern Germany. |
| 8 | **When and How Much** | Describe the number of times the intervention was delivered and over what period of time including the number of sessions, their schedule, and their duration, intensity, or dose | In the intervention manual it is recommended that the first four calls are on biweekly basis. After that the frequency is tailored to the needs of the patient. In fact participants received an average amount of 12.8 calls (SD=6.5) that lasted averagely 20.6 minutes (SD=8.4) each. |
| 9 | **Tailoring** | If the intervention was planned to be personalised, titrated or adapted, then describe what, why, when, and how | The intervention was tailored regarding the targeted chronic condition in different campaigns. The mental health and heart failure campaign were more specialised. Therefore the campaigns are prioritised if a participant was eligible for more than one:   1. Mental health campaign 2. Heart failure campaign 3. Chronic campaign   In the “Heart failure campaign” the intensity of the coaching depends on a score calculated based on social environment (living alone or with family, having nursing service or not), the number of hospital stays in the last 2 years, and the NYHA-status. The lonelier, the more often in hospital and the higher the impairment the more coaching the patient gets one call every 3 weeks instead of every 6 weeks).  In the mental health campaign and the chronic campaign, the frequency and the topics are tailored to the patient’s wishes and choices. |
| 10* | **Modifications** | If the intervention was modified during the course of the study, describe the changes (what, why, when, and how) | There was just one small modification during the course of the study: The intervention was carried out longer than expected. 76.2% of the participants received at least one more call after 24 months. In average all participants received 3.3 calls (SD= 5.06) after 24 months. This extension was done when patients stated that they needed more support. |
| 11 | **How well** | Planned: If intervention adherence or fidelity was assessed, describe how and by whom, and if any strategies were used to maintain or improve fidelity, describe them | The coaches were required to adhere to the manuals and its coaching components (see (3) *“What”* and (4) *”How”*). They also were obliged to document the coaching session in a software program called ProvidinCare®(29). Furthermore the coaches were supervised by two experienced members of the study management team. |
| 12* |  | Actual: If intervention adherence or fidelity was assessed, describe the extent to which the intervention was delivered as planned | The extent to which the coaches adhered to the manuals was assessed and certified by the supervisors and chief coaches of the two teams. This showed a high treatment integrity, the intervention was delivered as planned. |

Appendix 2: TIDieR Checklist.
