# Supplementary material for: Effectiveness of Telephone-Based Health Coaching for Patients with Chronic Conditions: A Randomised Controlled Trial
Source: PLoS One. 2016 Sep 15;11(9):e0161269. doi: 10.1371/journal.pone.0161269 (PMC5025178; doi:10.1371/journal.pone.0161269)
Supplement: S1 Table — Regression model to identify patients with a risk of rehospitalisation higher than 50%. (DOCX) [file pone.0161269.s004.docx]

| **Original regression model (German)** | **Explanation** |
| --- | --- |
| Z = -2,0519893680 |  |
| + (-0,0809947090 * GESCHLECHT) | Sex |
| + (0,0092434670 * ALTER_ID) | Age |
| + (0,1044337760 * KN_DMP) | Participation Disease Management Program |
| + (-0,0000028800 * JAHRESEINKOMMEN_EUR) | Annual income |
| + (0,1265342750 * KN_RECHTSKREIS West) | Zip code |
| + (0,0845667130 * NAME_REGIONSGRUNDTYP ländliche Räume) | Rural area |
| + (-0,3980221350 * NAME_REGIONSGRUNDTYP unbekannt) | Unknown area |
| + (-0,0398132050 * NAME_REGIONSGRUNDTYP verstädterte Räume) | Urbanized area |
| + (0,5827462930 * KN_COPD) | Chronic obstructive pulmonary disease |
| + (0,0944934290 * KN_VORHOF) | Atrial fibrillation |
| + (-0,0000112000 * KRKH_BETRAG) | Costs for hospital stays |
| + (0,1936613770 * ANZ_KRKH_FAELLE) | Number of stays in a hospital |
| + (0,0050709900 * ANZ_KRKH_TAGE) | Number of days in a hospital |
| + (-0,0765279800 * KN_HAT_FAVE) |  |
| **ICD-10 Codes** | |
| + (-0,3552527660 * ICD_A_) | ICD-10 Code A “Certain infectious and parasitic diseases” |
| + (0,1905810530 * ICD_C_) | ICD-10 Code C “Malignant neoplasms” |
| + (0,3004543060 * ICD_C17_) | ICD-10 Code C17 “Malignant neoplasm of small intestine” |
| + (-0,3356347620 * ICD_C34) | ICD-10 Code C34 “Malignant neoplasm of bronchus and lung” |
| + (0,1753173270 * ICD_C50) | ICD-10 Code C50 “Malignant neoplasm of breast” |
| + (-0,4285652680 * ICD_C77_) | ICD-10 Code C77 ”Secondary and unspecified malignant neoplasm of lymph nodes” |
| + (0,1718328810 * ICD_D37_) | ICD-10 Code D37 “Neoplasm of uncertain or unknown behaviour of oral cavity and digestive organs” |
| + (-0,1789026110 * ICD_D68) | ICD-10 Code D68 “Other coagulation defects” |
| + (-0,2357823430 * ICD_D69_) | ICD-10 Code D69 ” Purpura and other haemorrhagic conditions” |
| + (0,1015356540 * ICD_E03) | ICD-10 Code E03: “Other hypothyroidism” |
| + (0,1948678910 * ICD_E89) | ICD-10 Code E89: “Postprocedural endocrine and metabolic disorders, not elsewhere classified” |
| + (0,1186095830 * ICD_F_) | ICD-10 Code F: “Mental and behavioural disorders” |
| + (0,4052046880 * ICD_F10_) | ICD-10 Code F10: “Mental and behavioural disorders due to use of alcohol” |
| + (0,3098711410 * ICD_G20_) | ICD-10 Code G20: “Parkinson disease” |
| + (0,2422626770 * ICD_G47) | ICD-10 Code G47: “Sleep disorders“ |
| + (0,2620033780 * ICD_H_) | ICD-10 Code H: “Diseases of the eye and adnexa” |
| + (0,1711326070 * ICD_H40) | ICD-10 Code H40: “Glaucoma” |
| + (0,2013312550 * ICD_I05_) | ICD-10 Code I05: “Rheumatic mitral valve diseases” |
| + (-0,0416203260 * ICD_I10) | ICD-10 Code I10: “Essential (primary) hypertension” |
| + (-0,1696931810 * ICD_I21_) | ICD-10 Code I21: “Acute myocardial infarction” |
| + (0,1093786240 * ICD_I47_) | ICD-10 Code I47: “Paroxysmal tachycardia” |
| + (-0,1009491340 * ICD_I63_) | ICD-10 Code I63: “Cerebral infarction” |
| + (0,1406788420 * ICD_I65_) | ICD-10 Code I65: “Occlusion and stenosis of precerebral arteries, not resulting in cerebral infarction” |
| + (0,2752438400 * ICD_I70) | ICD-10 Code I70: “Atherosclerosis” |
| + (-0,1754652910 * ICD_I80_) | ICD-10 Code I 80: “Phlebitis and thrombophlebitis” |
| + (-0,1066716530 * ICD_J_) | ICD-10 Code J: “Diseases of the respiratory system” |
| + (-0,2318686350 * ICD_J15_) | ICD-10 Code J15: “Bacterial pneumonia, not elsewhere classified” |
| + (-0,4676865720 * ICD_J44) | ICD-10 Code J44: “Other chronic obstructive pulmonary disease” |
| + (-0,1411132400 * ICD_J69) | ICD-10 Code J69: “Pneumonitis due to solids and liquids” |
| + (0,1812455260 * ICD_K57) | ICD-10 Code K57: “Diverticular disease of intestine” |
| + (-0,2104040670 * ICD_L89_) | ICD-10 Code L89: “Decubitus ulcer and pressure area” |
| + (0,1479334910 * ICD_M_) | ICD-10 Code M: “Diseases of the musculoskeletal system and connective tissue” |
| + (0,2003576860 * ICD_M05_) | ICD-10 Code M05: “Seropositive rheumatoid arthritis” |
| + (0,1407979590 * ICD_M17) | ICD-10 Code M17: “Gonarthrosis [arthrosis of knee]” |
| + (0,1262794770 * ICD_M47_) | ICD-10 Code M47: “Spondylosis“ |
| + (0,1488120910 * ICD_M53_) | ICD-10 Code M53: “Other dorsopathies, not elsewhere classified” |
| + (-0,1583139160 * ICD_N_) | ICD-10 Code N: “Diseases of the genitourinary system” |
| + (0,1068904800 * ICD_N39) | ICD-10 Code N39: “Other disorders of urinary system” |
| + (-0,1569575650 * ICD_R_) | ICD-10 Code R: “Symptoms, signs and abnormal clinical and laboratory findings, not elsewhere classified” |
| + (0,2603303640 * ICD_T80_) | ICD-10 Code T80: “Complications following infusion, transfusion and therapeutic injection” |
| + (0,1463404920 * ICD_Z_) | ICD-10 Code Z: “Factors influencing health status and contact with health services” |
| + (0,2171833640 * ICD_Z85) | ICD-10 Code Z85: “Personal history of malignant neoplasm” |
| + (0,0847014290 * ICD_Z95) | ICD-10 Code Z95: “Presence of cardiac and vascular implants and grafts” |
| **Anatomical Therapeutic Chemical / Defined Daily Dose Classification** | |
| + (0,0961006060 * ATC_A02_) | ATC Code A02: “Drugs for acid related disorders” |
| + (-0,2207770970 * ATC_A06_) | ATC Code A06: “Drugs for constipation” |
| + (0,0645927940 * ATC_C01_) | ATC Code C01: “Cardiac therapy” |
| + (-0,1018113760 * ATC_C02_) | ATC Code C02: “Antihypertensives” |
| + (-0,0874644220 * ATC_C07_) | ATC Code C07: “Beta blocking agents” |
| + (-0,0570410220 * ATC_C07AB) | ATC Code C07A and B: “Beta blocking agents” and “Beta blocking agents and thiazides“ |
| + (-0,0683705510 * ATC_C08_) | ATC Code C08: “Calcium channel blockers” |
| + (-0,0599328530 * ATC_C10_) | ATC Code C10: “Lipid modifying agents” |
| + (0,0441687520 * ATC_J01_) | ATC Code J01: “Antibacterials for systemic use” |
| + (0,1145607160 * ATC_M01_) | ATC Code M01: Antiinflammatory and antirheumatic products” |
| + (0,0628801100 * ATC_N02_) | ATC Code N02: Analgesics” |
| + (0,1233529180 * ATC_N03_) | ATC Code N03: Antiepileptics” |
| + (0,0722214480 * ATC_N06_) | ATC Code N06: Psychoanaleptics“ |
| + (0,0543173310 * ANZAHL_ATC) | Amount of ACTs |
| + (0,0001353480 * MED_PREIS_APO_BRUTTO) | Brutto price of drugs |

Appendix 1: Regression model to identify patients with a risk of rehospitalisation higher than 50%.
