# Supplementary material for: Effectiveness of Telephone-Based Health Coaching for Patients with Chronic Conditions: A Randomised Controlled Trial
Source: PLoS One. 2016 Sep 15;11(9):e0161269. doi: 10.1371/journal.pone.0161269 (PMC5025178; doi:10.1371/journal.pone.0161269)
Supplement: S2 Table — Descriptives of coaching goals. (DOCX) [file pone.0161269.s005.docx]

| **Goal** | **Percentage** |
| --- | --- |
| Understanding of illness | 58.7 |
| Preventive examination colon cancer (occult blood testing) | 46.8 |
| Vaccination pneumococci | 44.7 |
| Preventive examination colon cancer (colonoscopy) | 42.8 |
| Self-monitoring of blood pressure | 32.6 |
| Preventive examination cervical cancer | 27.9 |
| Reducing multiple medication | 26.3 |
| Vaccination influenza | 25.1 |
| Preparation and discussion of physician visit | 21.2 |
| Preventive examination prostate cancer | 19.7 |
| Exercise increase | 19.6 |
| Weight reduction | 17.6 |
| Fluid and weight control | 13.6 |
| Development of individual action plan | 12.5 |
| Development of individual coaching goals | 12.1 |
| Preventive examination breast cancer (mammography) | 11.1 |
| Food record | 10.2 |
| Consultation with medical specialist (cardiologist) | 9.3 |
| Improving compliance | 9.1 |
| Development of an emergency plan | 8.6 |
| Enabling Shared Decision Making | 7.9 |
| Dietary change | 6.8 |
| Medication safety | 6.6 |
| Smoking cessation | 6.1 |
| Crisis management | 5 |

**Others:** Foot examination, coronary sports, relationship between physician and patient, check-up eyes, special medications and medication interactions (like ACE-inhibitors, beta blockers), stress management, fall prevention and many more were < 5%
