# Supplementary material for: Effectiveness of Telephone-Based Health Coaching for Patients with Chronic Conditions: A Randomised Controlled Trial
Source: PLoS One. 2016 Sep 15;11(9):e0161269. doi: 10.1371/journal.pone.0161269 (PMC5025178; doi:10.1371/journal.pone.0161269)
Supplement: S3 Table — Frequencies of diseases* as defined by the Elixhauser Index, at Baseline (Post-PSM), by campaign. (DOCX) [file pone.0161269.s006.docx]

**Frequencies of diseases* as defined by the Elixhauser Index, at Baseline (Post-PSM), by campaign**

|  | **Chronic campaign** | | **Heart failure campaign** | | **Mental health campaign** | | **All 3 campaigns** | |
| --- | --- | --- | --- | --- | --- | --- | --- | --- |
|  | **CG %** | **IG %** | **CG %** | **IG %** | **CG %** | **IG %** | **CG %** | **IG %** |
| **Congestive heart failure** | 26.70 | 24.77 | 99.56 | 99.41 | 0.00 | 0.99 | 33.65 | 32.01 |
| **Cardiac arrhythmias** | 40.51 | 38.85 | 71.70 | 71.01 | 10.48 | 8.91 | 42.89 | 41.34 |
| **Valvular disease** | 19.08 | 18.39 | 45.04 | 44.38 | 0.25 | 1.98 | 21.26 | 20.65 |
| **Pulmonary circulation disorders** | 5.63 | 6.08 | 19.55 | 20.41 | 0.00 | 0.00 | 6.94 | 7.42 |
| **Peripheral vascular disorders** | 27.14 | 27.50 | 31.14 | 27.22 | 0.99 | 2.97 | 26.73 | 26.68 |
| **Hypertension, uncomplicated** | 90.53 | 91.49 | 91.79 | 86.69 | 24.92 | 28.71 | 88.56 | 88.96 |
| **Hypertension, complicated** | 23.24 | 22.59 | 46.06 | 44.97 | 0.25 | 3.96 | 24.95 | 24.40 |
| **Paralysis** | 1.58 | 1.77 | 4.34 | 1.48 | 0.25 | 0.00 | 1.83 | 1.68 |
| **Other neurological disorders** | 4.64 | 5.49 | 3.85 | 7.10 | 5.78 | 4.95 | 4.59 | 5.65 |
| **Chronic pulmonary disease** | 36.43 | 37.34 | 44.45 | 44.08 | 21.37 | 16.83 | 36.80 | 37.40 |
| **Diabetes, uncomplicated** | 43.68 | 44.12 | 49.65 | 45.56 | 10.89 | 7.92 | 43.27 | 43.12 |
| **Diabetes, complicated** | 25.42 | 26.28 | 30.55 | 28.11 | 3.22 | 4.95 | 25.26 | 25.79 |
| **Hypothyroidism** | 20.50 | 20.35 | 20.39 | 20.71 | 26.07 | 9.90 | 20.66 | 20.05 |
| **Renal failure** | 13.13 | 13.97 | 38.73 | 39.94 | 0.99 | 0.99 | 15.48 | 16.34 |
| **Liver disease** | 18.21 | 19.24 | 20.46 | 17.75 | 5.53 | 4.95 | 18.04 | 18.62 |
| **Peptic ulcer disease excl. bleeding** | 3.96 | 3.54 | 3.72 | 4.73 | 1.73 | 0.99 | 3.87 | 3.59 |
| **AIDS/HIV** | 0.00 | 0.00 | 0.00 | 0.00 | 0.00 | 0.00 | 0.00 | 0.00 |
| **Lymphoma** | 0.34 | 0.29 | 0.00 | 0.59 | 0.00 | 0.00 | 0.29 | 0.32 |
| **Metastatic cancer** | 1.25 | 1.25 | 1.85 | 0.89 | 0.00 | 0.00 | 1.27 | 1.17 |
| **Solid tumor without metastasis** | 13.97 | 13.42 | 13.19 | 13.02 | 1.90 | 0.99 | 13.50 | 12.98 |
| **Rheumatoid arthritis/ CVD** | 14.46 | 14.04 | 6.02 | 9.76 | 2.56 | 3.96 | 13.18 | 13.26 |
| **Coagulopathy** | 8.37 | 8.04 | 14.92 | 15.09 | 2.72 | 0.00 | 8.89 | 8.53 |
| **Obesity** | 33.18 | 34.94 | 42.41 | 38.46 | 16.58 | 21.78 | 33.64 | 34.90 |
| **Weight loss** | 1.76 | 1.88 | 2.61 | 2.37 | 0.00 | 0.99 | 1.80 | 1.90 |
| **Fluid and electrolyte disorders** | 9.49 | 8.99 | 29.31 | 30.77 | 9.82 | 2.97 | 11.62 | 11.14 |
| **Blood loss anemia** | 0.88 | 0.66 | 2.51 | 0.89 | 0.00 | 0.00 | 1.02 | 0.67 |
| **Deficiency anemia** | 4.38 | 4.39 | 4.46 | 5.62 | 1.49 | 2.97 | 4.29 | 4.47 |
| **Alcohol abuse** | 2.36 | 2.25 | 4.49 | 3.55 | 8.09 | 8.91 | 2.77 | 2.60 |
| **Drug abuse** | 1.14 | 0.85 | 0.32 | 0.30 | 3.63 | 0.99 | 1.13 | 0.79 |
| **Psychoses** | 0.71 | 0.70 | 2.59 | 0.89 | 40.59 | 22.77 | 2.19 | 1.43 |
| **Depression** | 30.84 | 30.59 | 22.53 | 27.22 | 89.93 | 95.05 | 31.84 | 32.30 |

*Based on ICD diagnoses from inpatient treatment, outpatient treatment and inability to work. CG, control group; IG, intervention group
